# Musculoskeletal effects of 5 days of bed rest with and without locomotion replacement training

**DOI:** 10.1007/s00421-014-3045-0

**Published:** 2014-11-26

**Authors:** E. Mulder, G. Clément, D. Linnarsson, W. H. Paloski, F. P. Wuyts, J. Zange, P. Frings-Meuthen, B. Johannes, V. Shushakov, M. Grunewald, N. Maassen, J. Buehlmeier, J. Rittweger

**Affiliations:** 1 Institute of Aerospace Medicine, German Aerospace Center (DLR), Cologne, Germany; 2 International Space University, Strasbourg, France; 3 Department of Physiology and Pharmacology, Karolinska Institutet, Stockholm, Sweden; 4 Department of Health and Human Performance, University of Houston, Houston, USA; 5 University of Antwerp, Antwerp, Belgium; 6 Institute of Sports Medicine, Hannover Medical School, Hanover, Germany; 7 Department of Food and Nutrition Sciences, University of Bonn, Bonn, Germany

**Keywords:** Hypokinesia, Exercise countermeasure, Skeletal muscle, Bone markers, Fatigability

## Abstract

**Objectives:**

The present study evaluated the effectiveness of a short and versatile daily exercise regime, named locomotion replacement training (LRT), to maintain muscle size, isometric strength, power, and endurance capacity of the leg muscles following 5 days of head-down tilt (HDT) bed rest.

**Methods:**

10 male subjects (age 29.4 ± 5.9 years; height 178.8 ± 3.7 cm; body mass 77.7 ± 4.1 kg) performed, in random order, 5 days of 6° head-down tilt bed rest (BR) with no exercise (CON), or BR with daily 25 min of upright standing (STA) or LRT.

**Results:**

Knee extensor and plantar flexor cross-sectional area (CSA) were reduced by 2–3 % following bed rest (*P* < 0.01) for CON and STA, yet maintained for LRT. Knee extensor isometric strength (MVC) decreased by 8 % for CON (*P* < 0.05), was maintained for STA, and increased with 12 % for LRT (*P* < 0.05). Plantar flexor MVC remained unaltered during the study. Maximum jump height declined (~1.5 cm) for all conditions (*P* < 0.001). Neural activation and knee extensor fatigability did not change with bed rest. Bone resorption increased during BR and neither LRT nor STA was able to prevent or attenuate this increase.

**Conclusion:**

LRT was adequate to maintain muscle size and to even increase knee extensor MVC, but not muscle power and bone integrity, which likely requires more intense and/or longer exercise regimes. However, with only some variables showing significant changes, we conclude that 5 days of BR is an inadequate approach for countermeasure assessments.

## Introduction

Human space exploration over the last decades has identified major structural and physiological changes in a variety of organ systems due to weightlessness. The muscles and bones of the trunk and legs that are involved in posture and locomotion lose mass and strength in orbit (LeBlanc et al. 2000a, b, 2007). It is generally believed that alterations in the musculoskeletal system primarily result from physical unloading. Skeletal muscle no longer contracts with tension and, in consequence, the mechanical stimuli for bone remodeling processes are missing. As such, spaceflight-induced muscle atrophy and bone loss can be readily simulated on Earth by bed rest or dry immersion. The countermeasures currently employed during spaceflight, including exercise, have been proven not to fully prevent the adverse effects on muscle and bone (Cavanagh et al. 2010; Gopalakrishnan et al. 2010; LeBlanc et al. 2000). In view of future human explorations to Mars and asteroids, there is a need to develop more protective and more time-efficient countermeasures. To date, numerous bed rest studies have been dedicated to assess the efficacy of exercise, nutritional, and/or pharmacological interventions, using various methodological approaches (Pavy-Le Traon et al. 2007). The present study was part of a series of short-term bed rest studies organized by the European Space Agency (ESA) to validate countermeasures using the bed rest model of microgravity. The study as a whole assessed the efficacy of a short and versatile regime, named locomotion replacement training [LRT (Mulder et al. 2014). Unlike many other previously tested countermeasure schemes, the LRT encompassed various loading schemes (static and dynamic contractions both short and sustained, both unilateral and bilateral)] to mimic the variety of stimuli that are needed to maintain musculoskeletal and cardiovascular systems in daily life. With respect to the latter, it seems reasonable to suggest that alterations in the cardiovascular system and fluid shifts may affect the musculoskeletal system during actual spaceflight. Blood flow, for instance, depends on force and duration of muscle contraction and the arterio-venous pressure difference. Whereas leg muscles exercising in upright posture in 1 g can profit from the action of the so called muscle pump, the “bird leg” situation in space and the lack of hydrostatic pressure may cause an increased hypoxia during exercise, which may reduce the efficiency of exercise countermeasures in microgravity. The LRT regime was specifically designed to be implementable during centrifugation, and hence the study was performed with the expectation that the present results would be applicable to using the LRT scheme during centrifugation in real spaceflight. Typical everyday activities such as running and jumping on Earth, for instance, generate forces (and consequently stresses and strains) that are high enough for bone strength to be maintained (Frost 2003; Nikander et al. 2010). However, during space missions there is virtually an absence of forces with a high rate of force development (RFD) acting on muscles and bones, even when using exercise countermeasures extensively (Cavanagh et al. 2010). To overcome the lack of high stress and strain, a training regime allowing astronauts to perform reactive jumps should be implemented, as reactive jumps induce the highest ground reaction forces (GRF) and RFD (Ebben et al. 2010). We hypothesized that the short and versatile LRT regime could be a time-efficient countermeasure with compensatory effects on the cardiovascular and the musculoskeletal systems (Smith et al. 2003; Vernikos et al. 1996). The efficacy of the upright LRT scheme was compared to upright standing and to bed rest only using highly standardized measurements that are conducted in all ESA-organized bed rest studies since 2010. Vernikos et al. (1996) suggested that short-duration bed rest studies could serve as a reliable model for the rapid screening of preventative therapeutic treatments. Hence, in the present study we specifically evaluated the effectiveness of the applied LRT scheme to maintain muscle size, isometric strength, power, and endurance capacity of the leg muscles following 5 days of HDT bed rest. We additionally evaluated whether LRT would also prevent increased bone resorption markers, which can be already observed in the first days of bed rest (Baecker et al. 1985).

## Methods

### General design

The present 5-day study was part of a series of bed rest studies organized by the European Space Agency (ESA), starting with a short-term bed rest in preparation for more long-term studies. Details of the design have been presented elsewhere (Mulder et al. 2014). In short, a total of three bed rest campaigns were scheduled. Each campaign consisted of 5 days of baseline data collection (BDC-5 through BDC-1), 5 days of bed rest in 6° head-down tilt (HDT1 through HDT5), and 6 days of recovery (R + 0 through R + 5). The washout period between the end of campaign 1 and the start of campaign 2 was 50 days; the washout period between the end of campaign 2 and the start of campaign 3 was 94 days. Each subject randomly performed bed rest only (CON), bed rest with 25 min of daily upright standing (STA), or bed rest with 25 min of locomotion replacement training (LRT). In bed, the subjects maintained the 6° HDT for 24 h/day (except for 25 min in the LRT and STA interventions). The study design was approved by the Ethics Committee of the Northern Rhine medical association in Düsseldorf, Germany and was organized by the DLR Institute of Aerospace Medicine.

### Subjects

10 male subjects who had given their written consent completed the study. Baseline characteristics are provided in Table 1. One subject discontinued the study on BDC-3 of the first campaign and was instantly replaced by a backup volunteer. This subject performed nonetheless all experiments (including familiarization sessions) that were planned for BDC-5 and BDC-4.

**Table 1:** Subject characteristics at baseline

	Age (years)	Height (cm)	Body mass (kg)	Body fat (%)
CON	29.7 ± 6.0	178.8 ± 4.8	77.8 ± 4.8	18.8 ± 3.7
STA	29.6 ± 5.8	178.8 ± 4.8	78.1 ± 4.9	18.8 ± 3.3
LRT	29.6 ± 5.8	178.8 ± 4.8	78.0 ± 5.0	18.4 ± 4.1

Body fat (%) is based on whole-body dual energy X-ray absorptiometry (DEXA)

*CON* bed rest only, *STA* upright standing, *LRT* locomotion replacement training

### Diet

During the entire study, the subjects received a strictly controlled and individualized diet, and all meals were completely consumed. The individual energy intake (total energy expenditure, TEE) was calculated by multiplying resting metabolic rate (RMR), measured by indirect calorimetry (Deltatrac II MBH 200 metabolic monitor, Datex-Ohmeda) with a physical activity level of 1.4 (during ambulatory phase) and 1.1 (during HDT) for physical activity plus 10 % for diet-induced thermogenesis (DIT). 29.7 ± 0.2 % of the daily energy intake was consumed as fat; 54.9 ± 2.2 % as carbohydrates, and protein was taken in, in the amount of 1.21 ± 0.01 g/kgBM per day. The daily diet was also constant for calcium (1,085 ± 62 mg), potassium (3.9 ± 0.3 g), sodium (2.3 ± 0.1 mmol/kg BW), and water (50 mL/kgBM) intake. Additional fluid and energy intake was administered in the form of water and diluted-apple juice following physically demanding experiments to compensate for sweat and energy loss. To assess sweat loss, subjects were weighed before and after the MVC and cycle ergometry tests. Any loss in mass was assumed to be due to loss of sweat. Only the energetic cost of the cycle ergometry test was incorporated, for which the following formula was used: gross mechanical efficacy (%) = mechanical power [(W) × 0.01443 (kcal/W) × 100]/Total metabolic power input (kcal). Gross mechanical efficacy was fixated for all subjects at 23 % and hence the total metabolic power input could be calculated from the wattage and the duration of the various stages of the cycle ergometer test. Due to the absence of sunlight exposure, the subjects were daily supplemented with 1,000 IU (international units) of vitamin D.

### Interventions and control condition

#### Locomotion replacement training (LRT)

Subjects executed the upright 25-min LRT session daily during the HDT phase. This session consisted of a combination of heel raises, squats and hopping exercises in the upright position (see Mulder et al. (2014) for details). In brief, subjects performed three blocks: block one consisted of 20 bilateral heel raises, 20 squats (90°) and 4 sets of 6 reactive jumps; block two consisted of 2 × 12 unilateral heel raises, 12 deep squats (60°) and jumping as above. Block three consisted of 2 × 12 unilateral heel raises, shallow squatting (120°), and cross hopping and finished with a static squat (90°). One minute of upright pause was incorporated between blocks. A Smith Machine with fixed rails (PTS-1000 Dual Action Smith™ Cage, Hoist Fitness Systems, San Diego, USA) was used to guide the heel raise and squat exercises. Squats and heel raises were performed against body weight plus the additional weight of the barbell (15 kg). The heel raises were performed with straight knees and without ankle dorsiflexion. The shallow squats were performed continuously for 3 min. The reactive jumps and the cross hopping (left–right-left–right, etc.) exercises were performed without Smith Machine. The reactive jumps were performed with the ball of the foot (heels not touching the ground) at ~3 repetitions per second separated by 15-s rest every six jumps. Cross hopping was performed continuously for 3 min at a frequency of 1.3 repetitions per second. The duration of the exercises was unchanged during the study, except for the static squat, which increased from 45 s at HDT1 to 70 s at HDT5 for motivational purposes.

#### Standing (STA)

Though gravitational loading per se (i.e., standing) partially preserved orthostatic tolerance during bed rest (Vernikos et al. 1996), the general consensus is that ‘static loading’ is ineffective to maintain bone and muscle integrity [e.g., (Lanyon and Rubin 1984)]. The standing condition was implemented as an ‘active control condition’ to test whether the effects of LRT were related to the exercise per se, or related to the fact that the exercises were performed in the upright (i.e., gravitationally loaded) posture. For this purpose, each subject stood upright directly next to the bed for 25 min. Both feet were in contact with the floor during and any type of physical activity (e.g., heel raise, squatting or walking) was prohibited.

#### Control condition (CON)

Subjects remained in HDT 24 h/day for 5 days and refrained from any type of physical exercise and/or upright posture.

### Muscle size

The maximum CSA of knee extensor and plantar flexor muscles from the right limbs were assessed once before (BDC-2), and once following BR (R + 0) using Magnetic Resonance Imaging (Siemens Sonata scanner) at 1.5 Tesla using a spin echo sequence (TR = 28.00 ms, TE = 4.78 ms). Axial images with 3 mm (thigh) or 2 mm (lower leg) slice thickness were acquired with a matrix of 256 × 224 pixels of 1.0 × 1.0 mm pixel size. Subjects were positioned with their thighs in the horizontal plane, and foot restraints were used for fixation. To prevent fluid shifts from influencing CSA secondary to a change in body position, subjects remained supine for 30 min before imaging started. The knee extensor and plantar flexor muscle were each manually encircled by one operator blinded to both the session and intervention, and CSA was calculated using semi-automated SliceOmatic 4.3 software (Tomovision, Magog, Canada). Sliding averages of CSA values were calculated for three successive slides (Mulder et al. 2006) and the highest mean value was used as maximum CSA for further evaluation.

### Muscle function

The maximal voluntary contraction (MVC) of the knee extensor, and plantar flexor muscle groups was assessed as the highest attained torque value before (BDC-1) and following bed rest (R + 0). MVC measurements were obtained from the left leg using the Biodex-3 system (Biodex Medical Systems, Shirley, New York, USA). Care was taken to align the axis of rotation of the dynamometer with the respective joint axes. After individual adjustments, the fixed positions were maintained for seat, shin pad, and dynamometer axis positions during subsequent tests. Subjects were firmly strapped to the examining chair before the measurements started. Isometric knee extension and knee flexion MVCs were obtained at knee angles of 90, 80, and 70° from full knee extension (0°). Plantar flexion and dorsal flexion MVCs were obtained at angles of −10, 0, +10, +20, and +30° from ankle neutral position (0°). At each joint angle, which was assigned in random order, the subjects performed a 5–7 s maximum isometric extension followed by a maximum flexion after 30 s of rest, and 30 s thereafter by another pair of extension and flexion contractions until three complete sets of extension/flexion contractions were obtained. A 2-min rest was incorporated before proceeding to the next randomized joint angle. In this paper we present only data from the knee extensions and plantar flexions because electromyographic activity (see below) was recorded only from m. vastus lateralis and* m. gastrognemius medialis*. Subjects received loud verbal encouragement during the performance of the maximal contractions.

Knee extensor muscle fatigability was assessed before (BDC-1) and following BR (R+ 0) at a knee angle of 80°, using a 90-s sustained submaximal isometric contraction. The target torque was set at 50 % of the highest torque achieved in the knee extension isometric MVC test at 80° at the day of testing. Before each test, two to three practice contractions were performed until the subject managed to reach the visualized 50 % MVC target torque without difficulty. Following a 2-min rest, the subjects were instructed to quickly reach the target torque and maintain it for 90 s without interruptions. Verbal encouragement was provided to the subjects to reach the target torque until the finish time. As some subjects could not sustain the 90-s contraction without interruptions, the time to task failure was assessed as the time until the torque declined >5 % of the initial value for a period longer than 2 s.

Motor unit activity in the* m. vastus* laterals during knee extension and in the *m. gastrocnemius medialis* during plantar flexion was recorded with bipolar differential electromyography (EMG) using a Noraxon MyoSystem 1400A and Ag–AgCl surface electrodes directly connected with pre-amplifiers. Before applying the electrodes, the skin was shaved, cleaned and scrubbed with sandpaper. Skin–electrode resistance was checked for being lower than 10 kΩ and the skin was re-prepared if needed. The correct position of the electrodes was verified by M-wave assessments and this position was retained across the study using cutaneous ink marks. To also ensure consistency across campaigns, individual EMG templates (transparent plastic sheets with identified landmarks and electrode locations) were constructed during the initial pretest and subsequently used prior to all tests. Torque and EMG signals were digitized at 1,000 Hz using Noraxon software and processed by customized software. For each MVC trial, the peak torque value of a 0.5 s interval was assessed and EMG amplitude (RMS) was averaged for that time. The contraction that yielded the highest peak torque value at any of the angles was finally selected for comparisons. To discriminate between changes in RMS due to alterations in neural drive from changes due to alterations in peripheral factors, we normalized the RMS to the RMS of the M-wave (Arabadzhiev et al. 2010). RMS and median frequency (FM) during the knee extensor fatiguing contraction were assessed over the first 2 s and the 2 s immediately preceding task failure.

The countermovement jump test was assessed before (BDC-1) and following bed rest (R + 0). Subjects refrained from any type of exercise on the days of testing. Subjects stood on a ground reaction force plate (Leonardo, Novotec Medical GmbH, Pforzheim, Germany) with their hand on their hips. When stipulated by the Leonardo software, subjects flexed their knees and subsequently jumped as high as possible. During the jump, the hands remained on the hips. Trials were repeated when the subjects landed outside the platform, or had to be actively supported in maintaining balance following landing. The procedure was repeated until three valid trials had been acquired. The assessment of maximal force, maximal velocity and maximal jump height and countermovement depth (i.e., the lowering of the center of mass during the countermovement) was performed using the ground reaction forces, software provided by the manufacturer as well as customized software.

Before the actual functional testing procedures at BDC-1, each subject was familiarized with the equipment and the proper techniques during dedicated sessions scheduled at BDC-3. This familiarization session was identical in setup as the actual testing sessions, but the data are not included in the comparisons.

### Biological sample collection

Fasting blood samples were taken on days BDC-3, BDC-1; HDT2, and HDT5; and R + 1 and R + 5 in the supine or HDT position under standardized conditions at ~7:00 a.m. shortly after subjects awakened. Whole blood was centrifuged after coagulation (3,000 rpm, 4 °C, 10 min), and serum was distributed in small aliquots and immediately frozen at −80 °C until analysis. Urine was collected as 24-h urine pools on all study days from ±7:00 a.m. to ±7:00 a.m. on the following day. Single voids were stored under darkened and cooled conditions until final pooling into 24-h volumes. The subsequently obtained aliquots were stored at −20 °C.

### Laboratory methods

Serum concentrations of bone formation markers bAP and P1NP, as well as urinary bone resorption markers NTX and CTX, were determined with commercially available assays in the in-house laboratory of the Institute of Aerospace Medicine (bAP: Tandem R, Ostase, Hybritech, Liege, Belgium; PINP: Orion Diagnostica, Finland; NTX: Osteomark, Wampole Laboratories, Princeton, NJ; CTX: Crosslaps, Osteometer BioTech, Herlev, Denmark). Interassay and intraassay variations were as follows. Interassay were bAP, 8.8 %; PINP, 3.5 %; NTX, 4.0; CTX, 5.5 %; intraassay were bAP, 7.4 %; PINP, 3.5 %; NTX, 1.5 %; CTX, 2.5 %. Urinary calcium concentrations were analyzed in duplicate by flame photometry (EFOX 5053, Eppendorf, Germany).

Total urinary nitrogen was determined by highly sensitive chemiluminescence with a TNM-1 automated analyzer (Total Nitrogen Measuring Unit, Shimadzu, USA) and sample injector ASI-V (Shimadzu, USA). Within a series of control analyses performed each day using freshly prepared calibrators the coefficient of variation of this method was 1.00 % and the recovery was 103.68 %. Nitrogen balance was estimated as nitrogen intake (protein/6.25) minus urinary nitrogen excretion. Because nitrogen losses through skin and feces are very low and regarded as constant (Frings-Meuthen et al. 1985), these were not taken into account when calculating nitrogen balance.

### Statistics

In all parameters the effects of interventions (CON, STA, LRT) and sessions (BDC, HDT1-HDT5, R) were analyzed using Linear mixed effect (LME) models with sessions and intervention as fixed effects and subject ID as random effect. Variances were allowed to differ between participants, and LME models were optimized according to the Akaike information criterion [see p.353 and p.652 in (Crawley 2007)]. Data were box–cox transformed where indicated by test on normal distribution [Kolmogorov–Smirnov (SPSS) or Shapiro (R)], non-linear quantile–quantile plots or in case of heteroscedasticity. Initially, all HDT days, as well as the recovery days, were tested against the lumped BDC days. For bone resorption, day HDT1 was lumped with the BDC data. This was done because bone resorption is known to increase in the second day of bed rest. Hence, the first day of bed rest, from a bone physiology point of view, pertains to BDC rather than to HDT. The data of CSA were only obtained pre and post bed rest. Models were then further simplified in a step-wise manner when no significant intervention effects were found. First, data from all HDT days were lumped together, and so were data from all recovery days to yield the HDT phase and the REC phase. When there were still no significant effects, then STA and CON interventions were pooled to yield NO_LRT vs LRT comparisons. The last step of model simplification was the deletion intervention effects, so that pure phase effects were analyzed as the simplest model. Where ANOVA indicated significant effects, these were followed up with treatment contrasts with BDC as the reference. In addition GLMs were calculated to evaluate specific differences among certain sessions. Statistical analyses were carried out in SPSS v 20.0 and in the “R” statistical environment (version 2.9.2, www.r-project.org). Data are given as means and standard errors (SE) if not indicated otherwise. The level for statistical significance was set to *α* < 0.05.

## Results

### Muscle size and function

Knee extensor and plantar flexor muscle CSA, EMG amplitude, isometric strength data and optimal angles for the CON, STA and LRT interventions are shown in Table 2. Knee extensor size was reduced for CON (*P* < 0.001) and STA (*P* < 0.001), and maintained for LRT. Knee extensor MVC declined for CON (*P* < 0.05), was maintained for STA, and increased for LRT (*P* < 0.05). Plantar flexor size was reduced for CON (*P* < 0.01) and STA (*P* < 0.05), and maintained for LRT. The differences between responses were significant (i.e., intervention x bed rest interactions; all *P* < 0.05). Plantar flexor MVC remained unaltered during the study. No changes were seen in the respective maximum EMG amplitudes and optimal joint angles.

**Table 2:** Knee extensor and plantar flexor size and function for CON, STA, and LRT before (Pre) and after (Post) 5 days of bed rest

Variable	CON		STA		LRT	
	Pre	Post	Pre	Post	Pre	Post
Knee extensors						
max CSA (mm^2^)	7,835 ± 227	7,665 ± 218***	7,875 ± 231	7,669 ± 220***	7,785 ± 227	7,856 ± 253
MVC (Nm)	285.9 ± 10.3	261.3 ± 11.1*	270.6 ± 13.3	277.1 ± 11.3	265.4 ± 11.2	296.6 ± 12.9*
EMG_RMS_ (%)	2.26 ± 0.35	2.46 ± 0.60	2.80 ± 0.68	2.07 ± 0.34	2.22 ± 0.43	2.65 ± 0.74
Opt. angle (°)	74.0 ± 1.6	76.0 ± 1.6	76.0 ± 1.6	77.0 ± 1.5	75.0 ± 1.7	77.0 ± 2.1
Plantar flexors						
max CSA (mm^2^)	5,516 ± 164	5,384 ± 167**	5,578 ± 158	5,409 ± 150*	5,430 ± 153	5,464 ± 147
MVC (Nm)	188.2 ± 12.7	177.5 ± 11.5	191.7 ± 10.8	186.3 ± 11.1	189.8 ± 12.5	195.0 ± 8.8
EMG_RMS_ (%)	1.99 ± 0.47	2.18 ± 0.45	2.49 ± 0.56	2.78 ± 0.63	2.86 ± 1.09	3.15 ± 0.96
Opt. angle (°)	−10.0 ± 0.0	−10.0 ± 0.0	−9.0 ± 1.0	−10.0 ± 0.0	−9.0 ± 1.0	−10.0 ± 0.0

*CSA* cross-sectional area, *MVC* maximal voluntary isometric torque, *EMG*_*RMS*_ root-mean-squared amplitude of the electromyogram in percentage of M-Wave amplitude, *opt. angle* optimum joint angle that yielding the highest MVC

* *P* < 0.05, ** P < 0.01, *** P < 0.001 post different from pre

None of the indices related to knee extensor fatigability (Table 3) changed as a consequence of 5 days of bed rest in any of the intervention groups. Maximum jump height significantly declined (*P* < 0.001) without an effect of intervention. Jump power was not significantly reduced (*P* = 0.096). No effect of bed rest was observed for peak force. Countermovement depth was overall significantly lower for STA compared to CON (*P* < 0.01).

**Table 3:** Indices of knee extensor fatigability and counter movement jump performance from CON, STA, and LRT subjects before (Pre) and after (Post) 5 days of bed rest

Variable	CON		STA		LRT	
	Pre	Post	Pre	Post	Pre	Post
KE TTF (s)	57.3 ± 7.6	60.8 ± 6.8	62.6 ± 8.0	66.7 ± 7.2	65.1 ± 5.5	65.7 ± 5.9
KE ΔEMG_RMS_ (μV)	69.58 ± 16.95	82.11 ± 23.84	82.77 ± 25.89	106.46 ± 23.22	93.14 ± 17.58	81.01 ± 20.68
KE ΔEMS_MF_ (Hz)	−12.03 ± 2.32	−16.32 ± 3.36	−18.93 ± 3.76	−18.05 ± 3.18	−17.42 ± 3.09	−22.99 ± 3.07
CMJ max (cm)	39.87 ± 0.88	38.64 ± 0.97***	40.41 ± 1.08	38.56 ± 1.23***	39.88 ± 0.87	38.96 ± 0.89***
CMJ min (cm)	−29.00 ± 1.24	−29.00 ± 1.33	−32.69 ± 1.59	−30.80 ± 1.74	−29.76 ± 1.26	−29.74 ± 1.26
CMJ Pmax (W/kg)	44.29 ± 1.15	44.90 ± 1.20	44.17 ± 1.23	44.25 ± 1.15	44.24 ± 1.20	45.31 ± 1.22
CMJ Fmax (kN)	2.36 ± 0.04	2.42 ± 0.04	2.43 ± 0.04	2.40 ± 0.04	2.37 ± 0.05	2.42 ± 0.06

*KE* knee extensor, *TTF* time to task failure, Δ*EMG*_*RMS*_ the absolute change (final 2 s mean − initial 2 s mean) in root-mean-squared amplitude of the electromyogram during the fatiguing contraction, Δ*RMS*_*MF*_ the absolute change (final 2 s mean − initial 2 s mean) in median frequency of the electromyogram during the fatiguing contraction, *CMJ* countermovement jump; max, maximum jump height; min, countermovement depth, *Pmax* maximum Power normalized for body mass (kg), *Fmax* maximum Force

*** P < 0.001 post different from pre (data pooled across interventions)

### Nitrogen balance

**Fig. 1:**
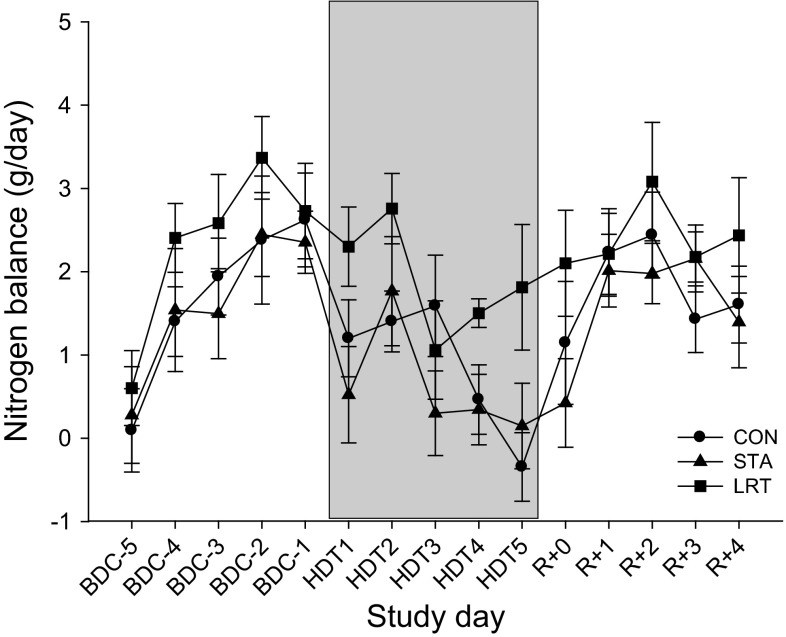
The day-to-day nitrogen balance (nutritional intake minus urinary excretion) during the study for the CON, STA and LRT interventions. Data are provided as mean ± SEM

The day-to-day absolute nitrogen balance is provided in Fig. 1 during the study for the CON, STA and LRT interventions. Data (mean ± SEM) represent the absolute balance, i.e., the difference between nutritional intake minus urinary excretion.

The per-phase-summed nitrogen balance was significantly lower for HDT, when compared to both BDC (*P* < 0.001) and R + (*P* < 0.001), without a significant difference between interventions. Mean data ± SEM for the CON, STA and LRT interventions are shown in Table 4.

**Table 4:** Mean Nitrogen balance values for CON, STA, and LRT during baseline (BDC), bed rest (HDT) and recovery (R)

Intervention	Absolute nitrogen balance (g) (cumulative intake − urinary excretion)		
	BDC	HDT	*R*+
CON	8.44 ± 1.98	4.31 ± 1.89***	8.85 ± 1.89
STA	8.11 ± 2.10	3.08 ± 2.41***	7.96 ± 1.92
LRT	11.68 ± 2.24	9.43 ± 1.67***	12.01 ± 2.49

Values are presented as mean cumulative sums ± SEM for each intervention for each study phase

*** P < 0.001 HDT different from BDC and R+ (data pooled across interventions)

### Markers of bone resorption

#### Urinary carboxy-terminal collagen crosslinks

**Fig. 2:**
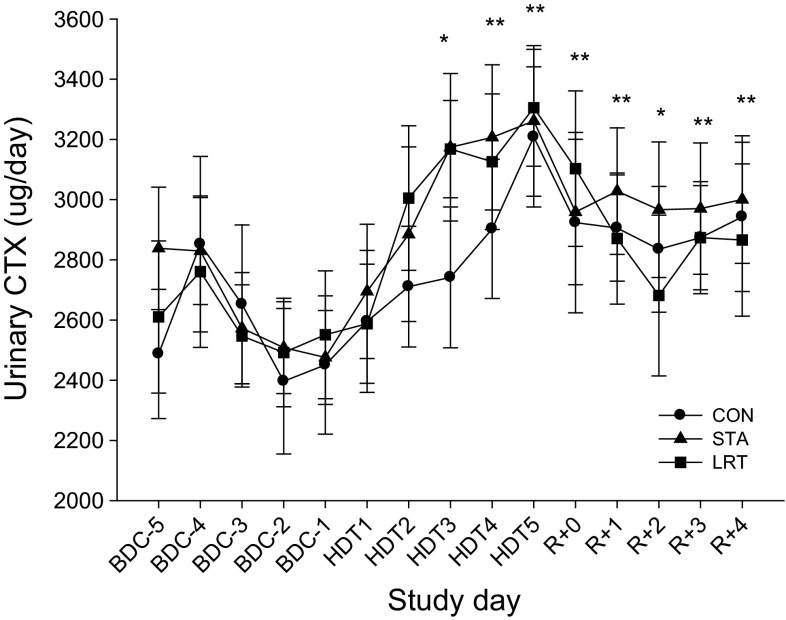
Time course of urinary bone resorption marker CTX throughout the study for the CON STA and LRT interventions. Data are provided as mean ± SEM. *P < 0.05, **P < 0.01 different from BDC-1

Taking BDC-1 as reference, there was a strong tendency that the UCTX levels (Fig. 2) were increased as early as HDT2 (*P* = 0.054); from HDT3 onwards UCTX levels were significantly increased (all *P* < 0.01, except for HDT3 and R + 2; *P* < 0.05). No differences were observed between interventions.

### Urinary amino-terminal collagen crosslinks

**Fig. 3:**
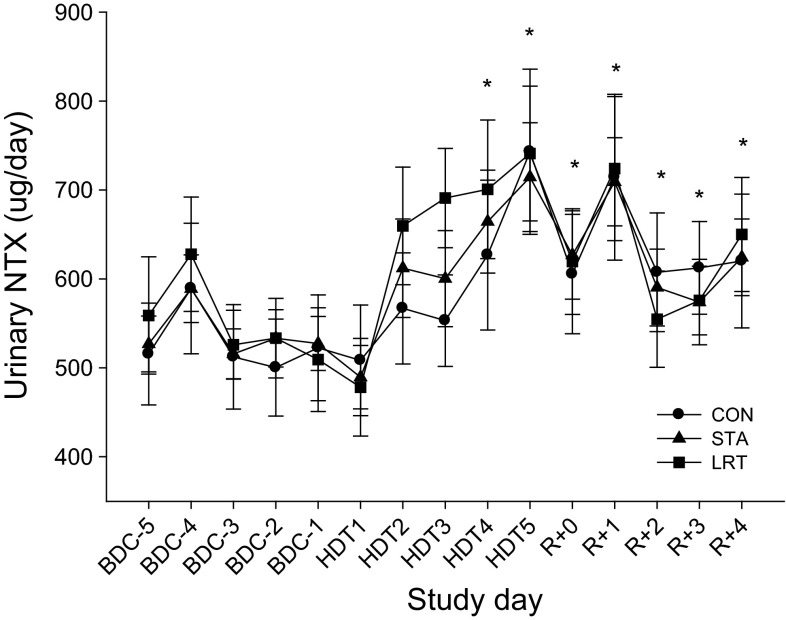
Time course of urinary bone resorption marker NTX throughout the study for the CON STA and LRT interventions. Data are provided as mean ± SEM. *P < 0.05

Taking BDC-1 as reference, UNTX levels (Fig. 3) were increased from HDT4 onwards (*P* < 0.05). No differences were observed between interventions.

### Markers of bone formation

#### Bone-specific alkaline phosphatase

Lumping the measurements per study phase (Table 5) revealed a significant effect between study phases (*P* < 0.001), without a difference between interventions. Mean bAP concentration was significantly higher during HDT compared to BDC (*P* < 0.01) and R+ (*P* < 0.05).

**Table 5:** Markers of bone formation for CON, STA, and LRT during baseline (BDC), bed rest (HDT) and recovery (R)

Bone marker	Intervention	BDC	HDT	*R*
bAP (μg/l)	CON	10.30 ± 0.76	10.99 ± 0.76	10.38 ± 0.81
	STA	10.07 ± 0.71	10.92 ± 0.75	10.66 ± 0.78
	LRT	10.42 ± 0.56	11.06 ± 0.63	10.59 ± 0.57
P1NP (μg/l)	CON	55.90 ± 2.48	59.37 ± 2.67	54.77 ± 1.78
	STA	55.03 ± 1.25	59.83 ± 1.37	56.09 ± 1.52
	LRT	53.69 ± 1.86	55.90 ± 1.73	53.71 ± 1.61

*bAP* bone-specific alkaline phosphatase, *P1NP* procollagen type 1 amino-terminal propeptide

#### Procollagen type 1 amino-terminal propeptide

Lumping the measurements per study phase (Table 5) revealed a significant effect of between the study phases (*P* < 0.01), without a difference between interventions. Mean P1NP concentration was significantly higher during HDT compared to BDC (*P* < 0.05) and R + (*P* < 0.05).

## Discussion

The novel finding of the present study was that a versatile, yet relatively mildly intense, upright training regime prevented a loss in knee extensor and plantar flexor muscle size, and even increased knee extensor strength following 5 days of HDT bed rest. Indices of muscular endurance, power, and muscle activation were not changed following bed rest and were not influenced by the countermeasure. Moreover, the training regime was not able to reduce bed rest-induced increased bone resorption during the first days of bed rest.

The bed rest model has been widely used to mimic the effects of spaceflight and test the efficacy of exercise, nutritional, and pharmacological interventions (Pavy-Le Traon et al. 2007). Although numerous bed rest studies from several weeks up to 1 year have been conducted, Vernikos et al. (1996) suggested that studies in the order of just a few days were sufficient for the rapid screening of novel preventive measures. The present findings indicate that 5 days of bed rest resulted in a loss of both the calf and the thigh CSA of approximately 2–3 %, which is in good agreement with the 3–6 % loss reported after 7–8 days of bed rest, spaceflight and cast immobilization (Crawley 2007; Ferrando et al. 1995; LeBlanc et al. 1995; Richter et al. 1985). Nonetheless, in light of previous findings (Vernikos et al. 1996; Zange et al. 2009) and in accordance with our expectations, daily 25-min upright standing did not prevent this loss in CSA. Indeed, given the variety of stimuli that act to maintain the musculoskeletal and cardiovascular systems in daily life, the present study investigated the effectiveness of a short-duration regime to maintain knee extensor and plantar flexor size [details elsewhere (Mulder et al. 2014)]. The present results are encouraging in that we found that with daily LRT sessions plantar flexor and knee extensor size were maintained following 5 days of bed rest. Previous studies had shown that the calf muscles were not preserved under conditions of bed rest, when trained three times per week (Alkner and Tesch 2004a, b; Gallagher et al. 2005; Mulder et al. 2009). In an attempt to compensate for the reduced responsiveness of the calf musculature, the present study incorporated daily sessions in which heel raise exercises, cross hopping and reactive jumps were performed to primarily target the calf muscles. Considering that the subjects exercised against body weight only, it seems that not only the load, but also the daily number of repetitions and the variety in loading are important for the maintenance of muscle size.

The present study incorporated a 30-min supine resting phase before the MRI examinations commenced. It is known that the transition to the horizontal position causes a rapid loss in blood volume of leg muscles, which reaches equilibrium after about 15 min. At the same time, muscle size is also lost by a slow shift in interstitial fluid, which can exceed 120 min until reaching equilibrium Berg et al. (1993). We limited the supine position to 30 min as we anticipated that any remaining changes thereafter would be too low to be detectable with MRI. In addition, since the MRI examinations were always performed with in the same order, the variations of volume differences by the different outflow of interstitial water between measurements are expected to be negligible. Hence, we assume that the muscle size changes seen in the present study were mostly attributable to a loss in protein content, but we acknowledge that changes in the ion homeostasis of the muscle fibers and changes in the extracellular matrix may also have played a role. Alterations in muscle volume by altered content in glycogen as less likely because of the strictly controlled isocaloric diet of the present study.

Parallel to muscle atrophy, prolonged immobilization, bed rest and conditions of spaceflight have also been shown to result in a negative nitrogen balance indicating increased protein catabolism (Frings-Meuthen et al. 1985; Stein et al. 1985). Our data show that significant nitrogen loss appears within 5 days of bed rest. The fact that net balance remained positive is explained by the fact that we did not consider nitrogen losses in feces and sweat. These losses would shift the entire balance to lower values, presumably leading to negative values during the HDT phase. However, more importantly for the present study is that we did not observe any difference between the control condition and the interventions. Hence, in contrast to the local assessment of muscle atrophy by means of MRI, the nitrogen balance reflects a change in total nitrogen gain/loss at the whole-body level. Apparently at this level, the efficacy of LRT to maintain body protein could not be established for this short-duration study.

Many studies, if not all, report that the atrophic process is associated with significant reductions in strength, which indicates that muscular atrophy is associated with the diminished force production capacity of unloaded muscles. Yet, since muscle strength is often more severely compromised than muscle size following unloading (Alkner and Tesch 2004; Narici et al. 2003), myofiber thinning may not be the sole contributor. This hampered performance can have several causes along the chain of intention-to-move until the activation and response of the contractile machinery in the muscle. Thus, factors within the muscle itself, but also factors in the motor control may contribute to the decrement in muscle performance. Indeed, some researchers have argued that impairments in the neural excitation of unloaded muscles are partly, or even primarily responsible for the observed strength decrements, particularly after short-duration unloading studies (Deschenes et al. 2002; Kawakami et al. 2001). In the present study, EMG amplitude was obtained to determine whether neural performance was diminished after 5 days of bed rest, for which we found no evidence. In fact, no direct change in neural activation, be it an increase of a decrease, could be discerned, whereas plantar flexor size was reduced for both CON and STA interventions, no changes were found in plantar flexor isometric strength in the present study. Furthermore, knee extensor CSA and maximum strength was maintained for STA, whereas knee extensor CSA was maintained, yet isometric strength increased for LRT. Hence if anything, neuromuscular control for the steady-state contractions seemed improved in the present study. Since subjects underwent three campaigns and in each campaign the baseline testing session was preceded by a practice session, we suggest that 5 days of bed rest were simply not enough to cause any measurable change in neural control for these well-practiced exercises.

Vertical jumps require a complex intra- and intermuscular co-ordination to translate the rotatory segment movements into linear acceleration of the body’s center of mass (Bobbert and van Soest 2001). It needs to be considered here that the vertical jump test has a very good reproducibility (Rittweger et al. 2004) and that isometric strength and vertical jump performance were repeatedly found to be dissociated after bed rest (Buehring et al. 1985; Rittweger et al. 2007). The present study has detected this kind of dissociation, with deteriorated jump performance and even partly increased muscle ‘strength’ after only 5 days. It is, therefore, likely that jumping performance requires muscle strength as well as co-ordination, and that co-ordination fades away more rapidly and is more difficult to recover (Rittweger et al. 2007) than muscle strength. The present findings show that jump height, albeit minutely, declined for all interventions. For STA and CON this might be expected, based on the changes in muscle size and strength, but for the LRT intervention hopping exercises and reactive jumps were integral components of the LRT scheme. On the other hand, the countermeasure jump was not specifically trained, which—in light of task specificity (Bobbert and van Soest 2001) might have contributed to the current findings. During space missions, there is a lack of high forces with a high rate of force development (RFD) acting on the muscles and bones, even when using the currently installed exercise countermeasures extensively (Cavanagh et al. 2010). Future studies should address what type of exercise is needed to prevent losses in lower extremity power following disuse. In daily life, elderly individuals who lack sufficient motor speed or possess poor lower extremity strength have an increased risk of fall-related bone fractures (Shigematsu et al. 2006).

Indices related to the endurance capacity of the knee extensor group did not change as a consequence of best rest. In the present study we used a 90-s, continuously held isometric contraction at 50 % of maximum knee extension strength at the day of testing. Electromyographic recordings served to locate any underlying causes of altered fatigability both at the central and at the peripheral level. Neither the time to failure, nor the used electromyographic indices changed following 5 days of bed rest. Previous studies examining the effect of disuse on local muscle fatigability have thus far yielded equivocal findings indeed. With respect to functional changes, some studies reported a reduced exercise capacity, i.e., a more fatigable muscle (Berg et al. 1993; Grichko et al. 1985; Mulder et al. 2007; Portero et al. 1996), an unaltered (Koryak 1996; Semmler et al. 2000; Weber et al. 2014) or even an increased muscle endurance capacity following real and simulated spaceflight (Clark et al. 2008; Deschenes et al. 2002; Semmler et al. 2000). Given the broad methodological spectrum of used testing regimes, it is tempting to speculate that the experimental paradigm and task specificity are the two single most important determinants in modulating changes in muscle fatigability following disuse.

Regarding bone metabolism, bone resorption increased, as expected [e.g., Baecker et al. (1985)], already on the second day of bed rest. The LRT or STA interventions were not able to prevent or even attenuate this increase. We already know that passive standing is quite ineffective for bone (Goemaere et al. 1994; Lanyon and Rubin 1984), which explains the ineffectiveness in the STA intervention. Gravitational loading of the tibia per se seems insufficient to maintain bone mass, whereas muscle contractions receive further importance preserving bone mass as shown by studies applying functional electrical stimulation or resistive exercise (Belanger et al. 2000; Rittweger et al. 2010). But although the mild-intense training prevented a loss in knee extensor and plantar flexor muscle size, and increased knee extensor strength, it was apparently ineffective for bone. Since bone adapts to mechanical stimuli that induce physiologic deformations of the bone (Frost 2003; Rubin and Lanyon 1987), we incorporated reactive jumps in the training protocol, as exercises with a high rate of force development (RFD) also seems to be important for bone homeostasis (Nikander et al. 2010), and reactive jumps have the highest ground reaction forces and RFD (Ebben et al. 2010). Apparently the forces, the kind of mechanical stimuli or the short-duration training seemed to be ineffective or too low to induce changes in bone metabolism in the present study. Bone formation was also not affected by the intervention. Previous studies have shown that exercise (treadmill running with application of lower body negative pressure, LBNP) can ameliorate the bed rest-induced increase in bone resorption markers (NTX, DPD) in men and women (Smith et al. 2003; Zwart et al. 2007). In contrast to the present 5-day study, the referred studies were 30 days in duration where bone resorption markers were elevated from pre-bed rest levels beginning on BR12/13. We did observe an unusual and at present unexplainable increase in bone formation during bed rest. Bed rest studies showed either decreased or unchanged bone formation levels (LeBlanc et al. 2007). The only but important difference between these studies and the present was the duration of bed rest, which was always longer than 5 days. Since the response time of the bone formation marker is usually much longer than the response time of bone resorption marker and more than just a few days (Watts 1999), and the effect on resorption markers in the cited papers was first visible after 2 weeks of bed rest, we conclude that 5 days of bed rest is an ineffective approach for testing countermeasures against changes in bone metabolism.

Inevitably, this study had some additional limitations that need to be addressed. As only some selected variables showing “significant” differences between conditions, the current study, even though it was a cross-over design, might have been underpowered. However, power calculations in our opinion, are most useful when there are well-defined end points and using a test with a well-established standard deviation. In the present multisystem bed rest study, there was a priori no given end point and the standard deviation of the responses was a priori not known. Hence, we accepted the experimental design established by the European Space Agency, based on our experience that an intervention that does not result in a statistically significant outcome in ten normal male subjects is probably not of physiological significance. The fact that only males were tested obviously limits the generalizability of our test results and warrants future studies with females. In conclusion, the present study showed that a relatively mildly intense, versatile training paradigm was adequate to maintain calf and thigh muscle size and sufficient to increase knee extensor isometric strength. Not only the load, but also the daily number of repetitions and the variety in loading seem to be important determinants for the maintenance of muscle size, and isometric strength. Maintenance of bone integrity requires more intense and/or longer exercise regimes than the one used in this study. However, with only some variables showing significant changes, we conclude that 5 days of BR is an inadequate approach for countermeasure assessments.

## Abbreviations

bAP: Bone-specific alkaline phosphatase
BDC: Baseline data collection
CON: No exercise
CSA: Cross-sectional area
CTX: Collagen type 1 cross-linked C-telopeptide
EMG: Electromyography
ESA: European Space Agency
FM: EMG median frequency
GRF: Ground reaction forces
HDT: Head-down tilt
IU: International units
LME: Linear mixed effect
LRT: Locomotion replacement training
MVC: Isometric strength
NTX: Collagen type 1 cross-linked N-telopeptide
P1NP: Procollagen type 1 N-terminal propeptide
R+: Recovery
RMR: Resting metabolic rate
RMS: EMG amplitude
RFD: Rate of force development
STA: Standing
TEE: Total energy expenditure
